# Hepatitis C virus screening practices and seropositivity among US veterans born during 1945 – 1965

**DOI:** 10.1186/1756-0500-7-449

**Published:** 2014-07-14

**Authors:** Emily J Cartwright, Christopher Rentsch, David Rimland

**Affiliations:** 1 Division of Infectious Diseases, Emory University School of Medicine, 49 Jesse Hill Dr, Atlanta, Georgia 30303, USA; 2 Veterans Affairs Medical Center, 1670 Clairmont Road, Decatur, Georgia 30033, USA; 3 Division of Infectious Diseases, Emory University School of Medicine, Veterans Affairs Medical Center, 1670 Clairmont Road, Decatur, Georgia 30033, USA

**Keywords:** Hepatitis C, Screening, Prevention & control

## Abstract

**Background:**

The Centers for Disease Control and Prevention (CDC) and the United States Preventive Services Task Force (USPSTF) recently augmented risk-based hepatitis C (HCV) screening guidelines with a recommendation to perform one-time screening in all persons born during 1945 – 1965, a birth cohort known to have a higher prevalence of HCV. We sought to estimate the proportion of veterans seen at the Atlanta VA Medical Center (AVAMC) who had ever been screened for HCV infection by birth year.

**Methods:**

We used an administrative database of all veterans seen at the AVAMC between January 1, 2011 and December 31, 2011, and a laboratory generated list of all HCV antibody tests and HCV RNA viral loads that were performed at the AVAMC to determine receipt of screening and HCV antibody positivity. Odds ratios and 95% confidence intervals were estimated using SAS version 9.2 (SAS institute, Cary, North Carolina).

**Results:**

HCV antibody testing had ever been performed on 48% (41,556) of the veterans seen in 2011; 10% of those tested had a positive antibody. Confirmatory viral loads were performed in 96% of those with a positive antibody screen. Those born during 1945 – 1965 were more likely to have a HCV antibody performed when compared with those born in other years (54% vs. 41%, odds ratio [OR] 1.70, 95% Confidence Interval [CI] 1.65-1.74). Among veterans ever tested for HCV antibody (n = 41,556), those born during 1945 – 1965 were 6 times more likely to have a positive HCV antibody (15% vs. 3%, OR 5.87, 95% CI 5.32-6.78), and 3 times more likely to have chronic HCV infection (76% vs. 50%, OR 3.25, 95% CI 2.65-4.00).

**Conclusions:**

Nearly half of the veterans seen in 2011 at the AVAMC had ever been tested for HCV infection. When examined by birth cohort, over half of the veterans born during 1945 – 1965 had been screened for HCV and 15% of those screened had a positive HCV antibody. Our findings confirm the increased prevalence of HCV infection in persons born during 1945 – 1965 as identified in the updated CDC and USPSTF recommendations.

## Background

**Figure 1:**
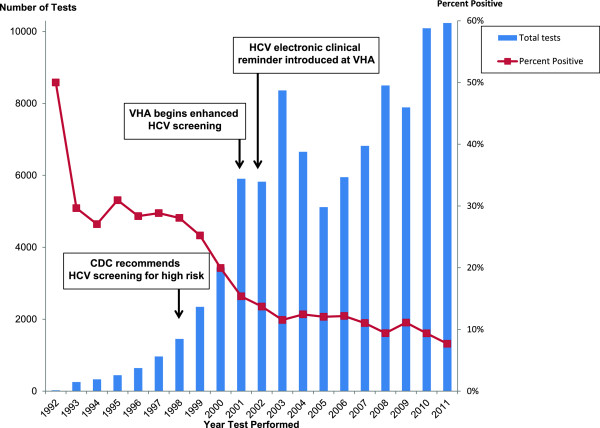
**Total number of hepatitis C antibody tests performed and percent of tests that are positive, by year — Atlanta VA Medical Center, Atlanta, GA, USA.** 1992 – 2011 (n = 91,240).

In 1990, serologic tests that detect antibodies to the hepatitis C virus (HCV) became commercially available in the United States. The Centers for Disease Control and Prevention (CDC) published guidelines on screening blood donors for HCV infection in 1991 and recommendations for HCV screening in persons with high risk behaviors in 1998 [1,2] (Figure 1). The US Veterans Health Administration (VHA) implemented HCV screening guidelines in 2001 that not only recommended screening veterans with the risk factors described in the CDC guidelines, but also screening any veteran with immoderate use of alcohol, a history of tattoos or repeated body piercing, intranasal cocaine use, multiple sexual partners, or Vietnam-era military service (i.e. dates of active military service between August 5, 1964 and May 7, 1975) [3]. In 2002, VHA introduced an electronic clinical reminder for HCV screening. In August 2012, CDC augmented risk-based HCV screening guidelines with a recommendation to perform one-time HCV screening in all persons born during 1945 – 1965, a birth cohort known to have a higher risk of having HCV infection [4,5]. In guidance published in June 2013, the United States Preventive Services Task Force also recommended one-time HCV screening of adults in this birth cohort [6]. Because this birth cohort overlaps with Vietnam-era veterans, we sought to estimate the proportion of veterans seen at the Atlanta VA Medical Center (AVAMC) who had ever been screened for HCV infection by birth year.

## Methods

We used an administrative database that contained the date of birth, gender, and race of veterans seen as inpatients or outpatients at the AVAMC between January 1, 2011 and December 31, 2011. All laboratory results were obtained from the AVAMC and included HCV antibody tests that were performed between 1992 and 2011 and all available HCV RNA viral loads. For veterans with more than one HCV antibody test, the HCV antibody status was considered positive if any HCV antibody test was positive. Any detectable value on either a quantitative or qualitative RNA viral load was considered positive. Continuous variables were compared using the Wilcoxon rank-sum test. Odds ratios and 95% confidence intervals were estimated using SAS version 9.2 (SAS institute, Cary, North Carolina). A *P* value of ≤ .05 was considered statistically significant. This study was approved by the Emory University Institutional Review Board and the VA Research and Development Committee. Informed consent was waived under a full HIPAA waiver.

## Results

From 1992 through 2011, 91,240 HCV antibody tests were completed on 67,539 veterans at the AVAMC (Figure 1). Before enhanced VHA screening efforts began in 2001, a median of 642 HCV antibody tests were done per year (interquartile range [IQR]: 329 – 1,451); since 2001, a median of 7,356 HCV antibody tests (IQR: 5,949 – 8,500) were done per year (*p* = 0.002).

In 2011, 87,144 veterans were seen at the AVAMC; data on age and sex was available in this database but information on race was largely missing (91% had a missing or unknown race) (Table 1). Over 50% of veterans seen were between 50 and 69 years of age. While 89% of all veterans seen in 2011 are male, when examined by birth cohort, men made up 73% of those born after 1965. HCV antibody testing had ever been performed on 48% (41,556) of the veterans (Table 2). Of those who had antibody testing, 49% (20,396) are African American, 37% (15,343) are Caucasian, 1% (390) are “other” race (includes Asian, Native Hawaiian, Pacific Islander, American Indian, or Alaska Native); race is unknown in 13% (5,427) (data not shown). HCV antibody was positive in 10% (4,107) of those tested (Table 2). Of those with a positive HCV antibody, confirmatory RNA viral loads were performed in 96% (3,944). Chronic hepatitis C (i.e., a positive HCV antibody and a detectable RNA viral load) was identified in 73% (3,004).

**Table 1:** Gender (by birth cohort), age, and race of veterans seen in 2011 — Atlanta VA Medical Center (n = 87,144)

	**No.**	**%**
**Male, overall**	77295	88.7
Male, born before 1945	23230	98.0
Male, born during 1945 - 1965	41834	89.6
Male, born after 1965	12231	72.9
**Age, years**		
<30	3446	4.0
30-49	18981	21.8
50-69	46560	53.4
≥ 70	18157	20.8
**Race**		
American Indian/Alaska Native	14	0.0
Asian	3	0.0
Black	7121	8.2
White	929	1.1
Unknown/Missing	78997	90.7
Hispanic	80	0.1

**Table 2:** Performance and positivity of hepatitis C antibody and RNA viral load tests for veterans seen in 2011, by birth cohort - Atlanta VA Medical Center (n=87,144)

	**HCV Ab performed no.**	**HCV Ab performed %**	**HCV Ab positive no.**	**HCV Ab positive %**	**Confirmatory RNA performed no.**	**Confirmatory RNA %**	**RNA positive no.**	RNA positive (chronic disease) %*
**Overall**	41556	47.7%	4107	9.9%	3944	96.0%	3004	73.1%
**< 1945 (N = 23702)**	8378	35.3%	335	4.0%	305	91.0%	189	56.4%
**1945 - 1965 (N = 46668)**	25097	53.8%	3644	14.5%	3516	96.5%	2775	76.2%
**> 1965 (N = 16774)**	8081	48.2%	128	1.6%	123	96.1%	40	31.3%

Note: *HCV* – Hepatitis C virus, *AB* – Antibody, *no*. - Number.

*Percent is calculated using the HCV Ab positive number as the denominator.

When the veterans seen in 2011 were classified by birth year, 27% were born before 1945, 54% were born during 1945 – 1965, and 19% were born after 1965. Among those born before 1945, 35% (8,378) were ever tested for HCV antibody; of those tested, 4% (335) were HCV antibody positive, and 56% (189) of those with a positive HCV antibody had confirmed, chronic HCV infection (Table 2). Among those born during 1945 – 1965, 54% (25,097) had ever been tested; 15% (3,644) were HCV antibody positive, and 76% (2,775) of those with a positive antibody had confirmed, chronic infection. In those born after 1965, 48% (8,081) had been tested and 2% (128) were HCV antibody positive; 31% (40) of those with a positive HCV antibody had confirmed chronic HCV viremia. Over 97% with chronic HCV viremia are male; among those born after 1965, 90% of those with chronic HCV viremia are male (data not shown). Those born during 1945 – 1965 were more likely to have a HCV antibody performed when compared with those born in other years (54% vs. 41%, odds ratio [OR] 1.70, 95% Confidence Interval [CI] 1.65-1.74). Among veterans ever tested for HCV antibody, those born during 1945 – 1965 were 6 times more likely to have a positive HCV antibody (15% vs. 3%, OR 5.87, 95% CI 5.32-6.78) and 3 times more likely to have confirmed, chronic HCV infection (76% vs. 50%, OR 3.25, 95% CI 2.65-4.00).

## Discussion

We found that nearly 50% of the veterans seen at the AVAMC in 2011 had received HCV antibody screening. Similarly, an analysis of National VHA HCV screening practices found that 53% of veterans with at least one outpatient visit at any VA clinic in 2011 had received HCV screening [7]. Civilian primary care settings report HCV screening rates of 1 – 8% [8-10] but interventions designed to enhance HCV screening have been shown to increase screening in high risk persons to 40% [5,10]. It is likely that the enhanced HCV screening recommendations for veterans and the electronic clinical reminder contributed to the higher HCV screening practices observed in our population and other VHA settings.

After accounting for untested veterans, the overall HCV prevalence among veterans seen at the AVAMC in 2011 is between 5% and 10%. While this estimate is comparable to published estimates at other VHA settings and the national VA estimate [7,11-14], it is much higher than the estimated HCV prevalence of 1.6% obtained from a national civilian survey [15]. Even among those born during 1945 – 1965, the HCV tested prevalence is higher in the veteran population (15%) compared with the civilian population (3%) [15]. Although the higher HCV prevalence in veterans has been well described, reasons for the higher burden are not fully understood and are likely multifactorial.

Veterans born during 1945 – 1965 were more likely to be screened for HCV, to have a positive HCV antibody, and to develop chronic HCV infection. The VHA recommendation to perform HCV screening in Vietnam-era veterans likely explains the higher HCV screening seen in those born during 1945 – 1965. Our finding confirms the higher HCV prevalence in those born during 1945 – 1965, supporting the birth cohort screening recommendations of CDC and USPSTF. While it is not fully understood why HCV prevalence is higher in this birth cohort, it has been hypothesized that it reflects incident HCV infections acquired through experimental intravenous illicit drug use during the 1970’s and 1980’s [16,17]. Additionally, higher HCV prevalence has been observed in similar birth cohorts outside of the United States, including in Scotland [18], England [19], and Cameroon [20]. Lastly, we found that those born during 1945 – 1965 were at increased risk of having chronic HCV infection (i.e. having a positive antibody and a positive viral load). Among those born after 1965, only 31% of those with a positive HCV antibody also had a positive viral load. There are two possible explanations for having a positive HCV antibody and a negative viral load; namely, a false positive HCV antibody or clearance of HCV viremia. While it is surprising that relatively few persons born after 1965 had evidence of chronic HCV infection, it is notable that there are more females in this cohort. Female sex has been associated with clearance of HCV infection in prospective studies of acute HCV [21-24]. While complex host and pathogen factors likely influence the development of chronic HCV infection, further exploration of the association between birth year and the development of chronic HCV infection is warranted.

Confirmatory HCV RNA viral loads were performed in 96% of the HCV antibody positive persons. This high confirmatory testing rate was also seen in an analysis of national VA data [7]. In contrast, surveillance for HCV infections from eight civilian US sites found that confirmatory RNA viral loads were only performed in 50% of positive HCV antibody tests [25]. By reflexively performing HCV RNA viral load testing on positive HCV antibody tests, the AVAMC testing practices are consistent with the current CDC guidelines which recommend RNA viral loads on all reactive HCV antibody tests [26].

Our analysis was limited to testing performed at the AVAMC and did not include testing done at other VA hospitals or testing done outside of VA system. However, test results from additional sources would likely only increase the screening estimates in our analysis. Because this analysis used administrative databases, information on HCV behavioral risk factors, medical comorbidities, and the rationale used by providers for HCV screening is not known. Information on race was missing for most veterans seen at the AVAMC in 2011. Other administrative databases from the AVAMC shows that 40% of veterans seen in 2011 are African American race, 40% Caucasian, and 2% “other” race; race was unknown in 18%. CDC and USPSTF recommend HCV screening for all persons born during 1945 – 1965, regardless of race. The veteran population in our analysis is older than the general population in the United States [http://www.census.gov/2010census/]. Lastly, because this analysis included a predominantly male population our findings may not be applicable in other settings.

## Conclusions

The enhanced screening efforts undertaken at the AVAMC in 2001 resulted in a significant increase in the annual number of HCV antibody tests performed. The diagnosis of chronic HCV infection allows for prevention interventions such as alcohol counseling, vaccination against hepatitis A and B, screening for advanced liver fibrosis, and referral for antiviral therapy. HCV antiviral management is rapidly evolving with the development of better tolerated and more efficacious therapies [27]. Achieving a sustained virologic response to antiviral therapy is known to significantly reduce the risk of liver failure [28], hepatocellular carcinoma [29], liver-related and all-cause mortality [30,31], and reduces the risk of further HCV transmission.

We found that many veterans born during 1945 – 1965 had been screened for HCV infection at the AVAMC even before the augmented CDC screening guidelines were published in 2012. However, given the high prevalence of disease in this birth cohort and the importance of HCV detection, continued screening practices that target this birth cohort are warranted.

## Abbreviations

VHA: Veterans health administration; HCV: Hepatitis C virus; RNA: Ribonucleic acid; HIPAA: Health insurance portability and accountability act; OR: Odds ratio; CI: Confidence interval.

## Competing interests

The authors declare that they have no competing interests.

## Authors’ contributions

EC conceived and designed the study, performed data abstraction and analysis, and drafted the manuscript. CR provided input on study design, data analysis, data interpretation, statistical testing, and provided critical review of the manuscript. DR assisted with study design, obtained data, and provided input on data analysis and interpretation, and critically reviewed the manuscript. All authors read and approved the final manuscript.
